# Supplementary material for: Calcified Neurocysticercosis: Demographic, Clinical, and Radiological Characteristics of a Large Hospital-Based Patient Cohort
Source: Pathogens. 2023 Dec 27;13(1):26. doi: 10.3390/pathogens13010026 (PMC10820744; doi:10.3390/pathogens13010026)
Supplement: Supplementary file 1 [file pathogens-13-00026-s001.zip › pathogens-2726511-supplementary.pdf]

Supplementary Table S1. Demographic, clinical, and radiological profiles of patients with calcified NCC, with and without a previous diagnosis of viable infection

Characteristics	Previous diagnosis of viable infection		*P*
	No (n = 242)	Yes (n = 282)	
Age at enrollment (years)			
Mean ± SD	41.5 ± 16.4	39.2 ± 14.0	0.091
Sex			
Female	154 (63.6)	141 (50.0)	0.002
Male	88 (36.4)	141 (50.0)	
Age at start of symptoms (years)			
Mean ± SD	30.7 ± 17.2	27.7 ± 14.9	0.038
Type of first symptom			
No symptoms	2 (0.4)	0 (0.0)	0.050
Headache	135 (55.8)	138 (48.9)	
Seizures	25 (10.3)	47 (16.7)	
Headache and seizures	77 (31.8)	97 (34.4)	
Previous seizures			
No	82 (33.9)	27 (9.6)	< 0.001
Yes	160 (66.1)	255 (90.4)	
Years since first seizure			
Median [IQR]	3 [1-11]	6 [2-14]	0.001
Number of previous seizures			
Median [IQR}	7 [3-27]	8 [3-25]	0.275
Seizure semiology			
Pure focal seizures	38 (23.8)	44 (17.3)	0.106
Focal to bilateral tonic-clonic seizures	122 (76.2)	211 (82.7)	
History of status epilepticus			
No	146 (91.8)	227 (89.4)	0.412
Yes	13 (8.2)	27 (10.6)	
ASM			
No	21 (13.2)	26 (10.6)	0.418
Yes	138 (86.8)	220 (89.4)	
Seizure-free time before enrollment (months)			
Median [IQR]	2 [0-12]	12 [2-34]	< 0.001
Previous neurosurgery			
No	235 (97.1)	243 (86.2)	< 0.001
Yes	7 (2.9)	39 (13.8)	
Previous diagnosis of taeniasis			
No	207 (91.2)	221 (81.3)	0.002
Yes	20 (8.8)	51 (18.7)	

Family history of seizures			
No	166 (89.4)	203 (74.6)	0.703
Yes	61 (10.6)	69 (25.4)	
Family history of NCC			
No	203 (89.4)	242 (89.0)	0.870
Yes	24 (10.6)	30 (11.0)	
Baseline EEG results			
Normal	221 (94.8)	271 (98.2)	0.037
Abnormal	12 (5.2)	5 (1.8)	
EITB results (number of antibody bands)			
Median [IQR]	1 [0-3]	3 [2-5]	< 0.001
Number of calcifications on brain CT scan			
Median [IQR]	2 [1-5]	3 [1-9]	< 0.001
1	98 (40.5)	78 (27.7)	< 0.001
2 to 10	119 (49.2)	143 (50.7)	
+10	25 (10.3)	61 (21.6)	

Abbreviations: NCC (neurocysticercosis); SD (standard deviation); IQR (interquartile range); EEG (Electroencephalogram); EITB (Enzyme-linked Immunoelectrotransfer Blot); ASM (Anti-seizure medication); CT (computed tomography)

Supplementary Table S2. Clinical profiles of patients with calcified NCC, with single and multiple lesions.

Characteristics	Number of calcified lesions		*P*
	Single lesions (n = 176)	Multiple lesions (n = 348)	
Age at start of symptoms (years)			
Mean ± SD	28.7 ± 13.9	29.3 ± 17.1	0.651
Type of first symptom			
No symptoms	0 (0.0)	1 (0.3)	0.695
Headache	95 (54.0)	178 (51.2)	
Seizures	61 (34.7)	113 (32.5)	
Headache and seizures	19 (10.8)	53 (15.2)	
Previous seizures			
No	47 (26.7)	62 (17.8)	0.018
Yes	129 (73.3)	286 (82.2)	
Years since first seizure			
Median [IQR]	3 [1-10]	5 [2-14]	0.047
Number of previous seizures			
Median [IQR}	6 [3-29]	8 [3-32]	0.370
Seizure semiology			
Pure focal seizures	30 (23.3)	52 (18.2)	0.230
Focal to bilateral tonic-clonic seizures	99 (76.7)	234 (81.8)	
History of status epilepticus			
No	116 (89.9)	257 (90.5)	0.856
Yes	13 (10.1)	27 (9.5)	
ASM			
No	17 (13.2)	30 (10.9)	0.499
Yes	112 (86.8)	246 (89.1)	
Seizure-free time before enrollment (months)			
Median [IQR]	4 [0-20]	7 [1-31]	0.071

Abbreviations: NCC (neurocysticercosis); SD (standard deviation); IQR (interquartile range); EEG (Electroencephalogram); EITB (Enzyme-linked Immunoelectrotransfer Blot); ASM (Anti-seizure medication); CT (computed tomography)

Supplementary Table S3. Detailed description of EEG in patients with calcified NCC and abnormal EEG results at baseline (n = 17)

Id	Description of EEG results
1	Generalized cortical slowing with paroxysmal discharges
2	Focalized cortical slowing in the right hemisphere with paroxysmal discharges
3	Cortical dysfunction in the right frontal lobe
4	Generalized cortical slowing with interictal epileptic discharges
5	Generalized cortical slowing with paroxysmal discharges
6	Focalized cortical slowing in the left frontal lobe with paroxysmal discharges
7	Focalized cortical slowing in the left hemisphere with paroxysmal discharges
8	Focalized cortical slowing in the right hemisphere with predominance of the frontal lobe
9	Generalized cortical slowing with predominance of the right hemisphere and with paroxysmal discharges
10	Focalized cortical-subcortical slowing in the left hemisphere with non-paroxysmal discharges
11	Generalized cortical-subcortical slowing with non-paroxysmal discharges
12	Intermittent cortical-subcortical slowing in the right hemisphere
13	Intermittent diffuse cortical-subcortical slowing with non-paroxysmal discharges
14	Diffuse cortical-subcortical slowing with non-paroxysmal discharges
15	Intermittent cortical-subcortical slowing with non-paroxysmal discharges
16	Intermittent generalized bilateral theta activity
17	Intermittent generalized theta activity with bifrontal sharp waves suggesting abnormal epileptiform activity

Abbreviations: NCC (neurocysticercosis); EEG (electroencephalogram)
